# Photoneutron spectrometry by novel multi-directional spherical neutron spectrometry system

**DOI:** 10.1038/s41598-021-81529-5

**Published:** 2021-02-05

**Authors:** Mehdi Sohrabi, Amir Hakimi

**Affiliations:** Health Physics and Dosimetry Research Laboratory, Department of Energy Engineering and Physics, Amirkabir University of Technology, Tehran, Iran

**Keywords:** Particle physics, Medical research

## Abstract

Neutron spectrometry in science and technology applications in general and accurate exotic photoneutron (PN) dosimetry of cancer patients undergoing high-dose high-energy X-rays therapy in medical accelerators in particular is of vital need. In this study, a novel passive multi-directional multi-detector neutron spectrometry system was developed and home-made using 6 polycarbonate/^10^B detectors on 6 sides of polyethylene (PE) cubes used bare and also embedded at center of PE spheres of 8 different diameters. The system provided well-resolved unfolded directional PN spectra showing thermal and fast PN peaks of 6 sides and mean spectrum in 5 field sizes at isocenter and other locations in 18 MV Siemens ONCOR medical linear accelerator bunker. The neutron spectrometry system developed has unique characteristics such as being simple, efficient, low cost, practical, and insensitive to low-LET radiation with well-resolved directional and mean spectra easily applicable in medicine, health, environment, science and technology in developing and developed laboratories.

## Introduction

Neutron spectrometry is of vital need in many areas of health, medicine, environment, science and technology, in particular for accurate exotic photoneutron (PN) dosimetry of cancer patients undergoing high-dose high-energy X-ray therapy in medical linear accelerators for estimating undesired second primary cancer risks of organs^1,2^. Albeit the complexity, some neutron spectrometry methods have been advanced such as proton recoil method^3^, time of flight measurements^4^, threshold detectors^5^, ^3^He nested neutron spectrometer^2^, ultra-thin 3D silicon sensors^6^ and multi-sphere methods. In particular, Bonner-type polyethylene (PE) spheres coupled with embedded thermal neutron detectors such as activation detectors^2,7–9^; BF_3_ or ^3^He proportional counters^2,10–12^; CR-39/^10^B^13–16^; bubble detectors^17–19^; paired LiF TLDs^20,21^; and thermal neutron pulsed detectors^22^, have been practical and well-adapted methods.

Due to neutron-weighting-factor dependence on neutron energy^23^ and need to advance scientific developments, determination of directional neutron spectrum due to neutron scattering in the surrounding environmental media is of vital need. In order to better justify this need, two directional dependencies in neutron detection and spectrometry are defined here:

1. Inherent directional dependence of a thermal neutron detector embedded in a PE sphere, and
2. Geometric directional dependence of the neutron spectrometry system which is affected by its geometry and surrounding environmental media at the spectrometry location.

The geometric directional dependence is extremely important in accurate neutron dosimetry of workers in work environments and of patients undergoing high energy X-ray therapy or possibly neutron therapy. Some of the reasons may include:

- Radiobiological effects of neutrons which depend on neutron energy being itself highly affected by directional dependence of the system and surrounding media,
- Accurate determination of individual dose equivalents in a work environment requiring accurate directional neutron workplace monitoring, and
- Accurate determination of patients’ fast, epithermal and thermal PN doses separately and as sum value for estimating the second primary cancer risks of organs^1^.

To fulfill such needs, some directional neutron spectrometry methods have been advanced such as: superheated emulsions at the center of one single 30 cm diameter nylon-6 moderating-sphere with a telescope-design wherein the detector views a narrow solid angle of about 1/6 steradians from an Am-Be source^19^; multiple thermal neutron pulsed detectors in one moderating sphere exposed externally to Am-Be neutrons from different directions^22^; three orthogonal ^3^He tubes used inside a single high-density PE sphere exposed to Am-Be neutrons^12^, and design of an ultra-sensitive single cylindrical moderator directional neutron spectrometer based on seven ^3^He detectors applied to low-fluence cosmic-ray-induced neutrons at ground level^24^. These advances are indeed strong supports to vital need to a simple passive multi-directional neutron spectrometry system.

In fact, a neutron spectrometry system should have a detection system with high sensitivity to neutrons, no sensitivity to low-LET radiation (x, γ and β), simplicity in particular in operation, post-exposure time independence, easy to be home-made, wide applications when established, and of course capability to determine directional spectrum. In this context, a passive multi-directional multi-detector neutron spherical spectrometry system was hypothesized, designed, and developed in this study to fulfil such requirements. The neutron spectrometry system developed was then successfully applied to an exotic application; i.e. PN spectrometry in high-dose high-energy X-rays at the isocenter and some locations in the radiotherapy bunker of a Siemens ONCOR 18 MV X-ray medical linear accelerator (here after called medical accelerator). The detection method is based on applying the “Sohrabi neutron dosimetry methods” using polycarbonate track detectors (PCTD)/^10^B thermal neutron detector when PCTDs are processed by electrochemical etching (ECE) methods^25–30^. The directional-sensitive system is based on a PE cube having one PCTD/^10^B at each of its six sides. The PE cube is used either as bare, with no PE sphere, or embedded at the center of 8 Bonner-type PE spheres of different diameters. This design provides a potential to determine neutron spectrum of each of 6 sides of the PE cube and in turn to obtain a mean neutron spectrum of the point where neutron spectrometry is made.

Having said the above, it is the purpose of this study to;

1. Introduce a novel passive multi-directional multi-detector spherical neutron spectrometry system which applies 6 PCTD/^10^B detectors on 6 sides of a PE cube used either as bare or embedded at center of 8 PE spheres of different diameters,
2. Apply the developed system for directional PN spectrometry in an exotic high-dose rate 18 MV X-rays in a medical accelerator, and
3. Determine and report directional PN spectra at 5 different field sizes and locations such as at the isocenter, at 50 cm from the isocenter and at the maze-room junction of the radiotherapy bunker, in order to demonstrate its potential for other normal applications in medicine, health, environment, science and technology.

## Experiments and methods

### Passive multi-directional multi-detector spherical neutron spectrometry system

A passive multi-directional multi-detector spherical neutron spectrometry system was designed, home-made and applied in PN spectrometry in high-dose high-energy X-ray beam and other locations of a medical accelerator bunker; which is under extreme and exotic conditions of having a very low PN fuence in the presence of high-dose rate 18 MV X-rays. The spherical neutron spectrometry system consists of three main components including:

1. A base PCTD/^10^B thermal neutron detector,
2. A central PE cube holding 6 PCTD/^10^B thermal neutron detectors on its 6 sides, and
3. Eight PE spheres of 2, 3, 4, 5, 6, 8, 10 and 12 inch diameters having a hollow cube carved at their centers in order to embed the PE cube.

#### Base PCTD/^10^B detector

The PCTD/^10^B detector has been recently applied for extremely low-level PN dosimetry in 6 MV X-ray beams^31^, in 18 MV X-ray beams^1,5,7, 8,17,20,32,33^, and in a plasma focus device filled with deuterium gas^29^. PCTDs are highly sensitive to 1.47 MeV alpha particles through ^10^B(n_th_,α)^7^Li reaction **(**high cross section of 3840 barn), with near 100% efficiency when in contact with enriched ^10^B converter after processed by an ECE method^25–30^. It should be mentioned that PCTDs basically detect fast neutrons with energy > 1 MeV through fast-neutron-induced recoil particles by elastic scattering with hydrogen, carbon, and oxygen atoms. In particular, PCTD/^10^B detectors when decorated with or without cadmium can be used in neutron dosimetry with minimum detection levels of ~ 1.0 mSv, ~ 0.014 mSv and ~ 0.016 mSv for fast, epithermal and thermal neutrons respectively^34,35^.

After the necessary exposures were made, the PCTDs were removed from the spectrometry system and processed by the ECE method. Then mean track densities were determined by counting the alpha tracks under a light microscope. Accordingly, thermal PN fluences were obtained by converting a mean track density ± SD (tracks.cm^−2^) of each PCTD/^10^B detector to fluence by using relevant conversion factors.

#### Central polyethylene cube

**Figure 1:**
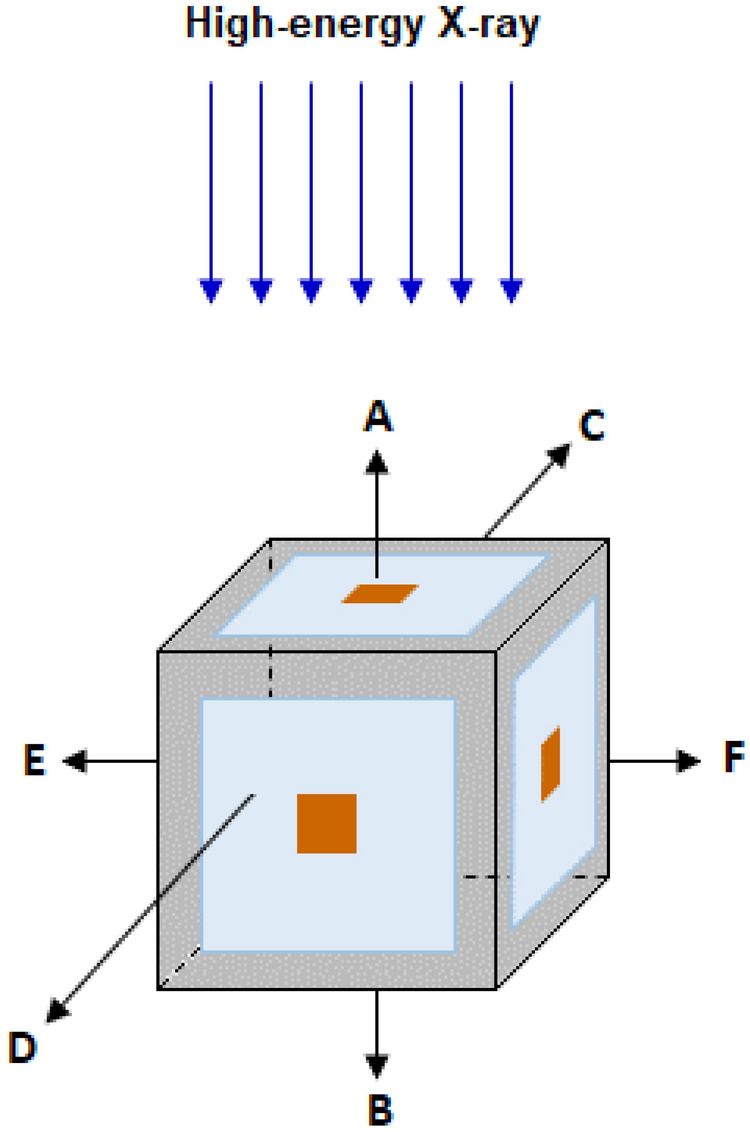
Schematic design of a PE cube with 6 PCTD/^10^B detectors on sides A, B, C, D, E and F sides of the cube.

In order to compensate for the inherent detector directional dependence and the geometric system directional dependence of the neutron spectrometry system, six PCTD/^10^B detectors were installed on 6 sides of a 4 cm × 4 cm × 4 cm PE cube. One PE cube was used as bare, with no PE sphere, and 8 PE cubes were embedded at the center of 8 PE spheres of different diameters. This design makes the PE sphere system to multi-directionally respond to neutrons coming from the main beam and from 5 other directions. Figure 1 shows schematic design of a PE cube with 6 PCTD/^10^B detectors on sides A, B, C, D, E, and F of the cube.

#### Polyethylene moderating spheres

The basic neutron energy thermalizing medium of this neutron spectrometry system is the PE spheres of different diameters used. The rationale in using PE material (C_2_H_4_)_n_ is that fast neutrons by interacting through elastic scattering with the atom constituents in particular hydrogen atoms are thermalized and detected by each side thermal neutron detector. As the size of the PE sphere increases, more energetic neutrons are thermalized and detected. Therefore, having PE spheres of different diameters in an increasing order allows detecting fast neutrons with more energy ranges.

**Figure 2:**
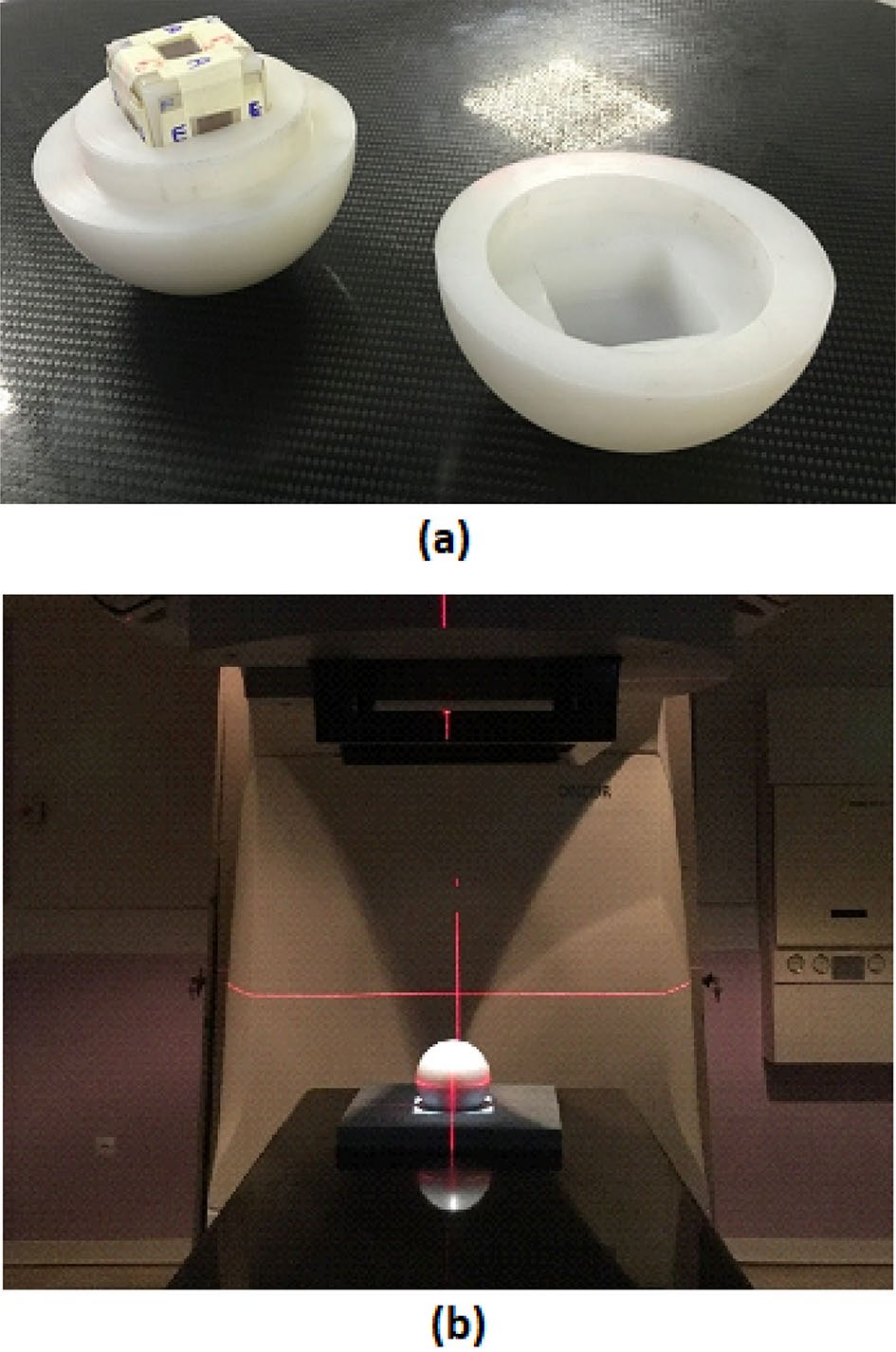
(**a**) Halves of a PE sphere with a PE cube embedded (with 6 PCTD/^10^B detectors on the sides); and (**b**) the assembled PE sphere with embedded PE cube as placed at the isocenter of the medical accelerator beam to be exposed.

In this study, a set of 8 specially-designed PE spheres (ρ = 0.9 g cm^−3^) with 2, 3, 4, 5, 6, 8, 10 and 12 inch diameters were home-made and used. Each PE sphere consists of two halves with a hexagonal cavity carved at the center to embed the PE cube with 6 PCTD/^10^B when assembled together. Figure 2a,b shows a PE sphere with a PE cube having 6 PCTD/^10^B detectors on sides as embedded; (a) in the hexagonal cavity of a half sphere, and (b) at the center of a PE sphere placed at the isocenter of the medical accelerator beam to be exposed. In order to get the full neutron spectrum at each location, each of the 8 spheres has been independently placed one by one at the same location and exposed to equal 18 MV X-ray doses of 5 Gy. In order to block external thermal neutrons from entering the PE sphere, each PE sphere was covered by a cadmium sheet with a thickness of 0.5 mm optimized in a previous study^31^.

#### Electrochemical etching of polycarbonate detectors

Each PCTD used in a PCTD/^10^B detector is 3.1 cm × 3.1 cm in size and 250 µm thick cut from a larger polycarbonate sheet. Polycarbonate sheets are masked on both sides to prevent any scratches and are commercially available in common plastic markets. The masks are removed when placed on the PE cube to be exposed. The PCTDs after exposure to PNs were processed by a high frequency–high voltage (HF–HV) ECE method under optimized ECE field conditions of 2 kHz–32 kV cm^−1^ in PEW solution (15 g KOH + 40 g C_2_H_5_OH + 45 g H_2_O) at 26 ± 1 °C for 3 hours^25–30^.

#### Medical accelerator bunker

**Figure 3:**
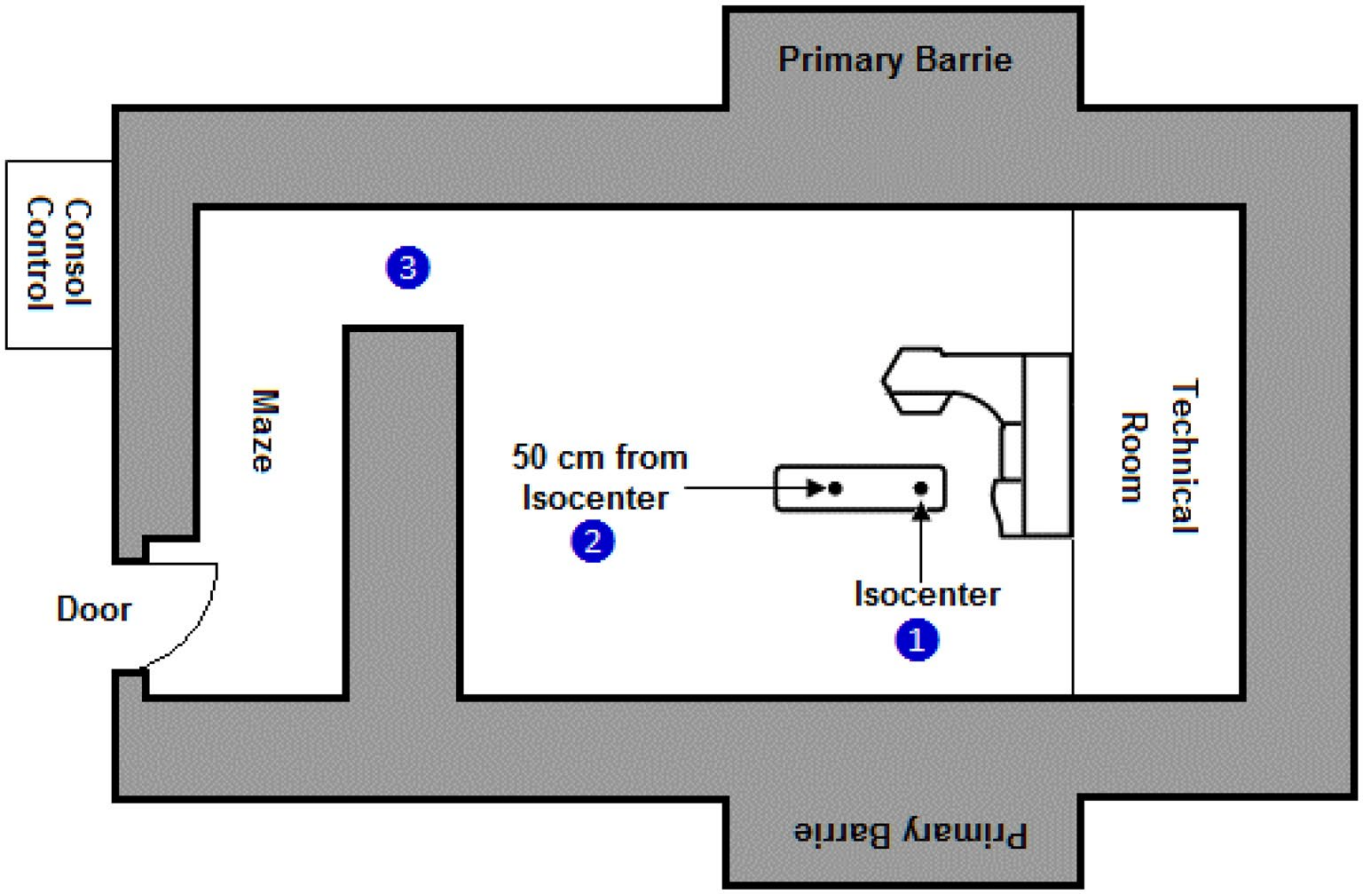
Three PN spectrometry measurement locations shown inside the radiotherapy treatment bunker of the medical accelerator (darker shaded regions are high density concrete).

PN spectrometry studies were performed in the 18 MV X-ray beam and two other locations of a Siemens ONCOR dual energy medical linear accelerator bunker (Siemens Healthcare, Erlangen, Germany). The medical accelerator is equipped with double-focused multi-leaf collimators consisting of 41 leaves of 1 cm resolution at the isocenter. Determination of PN spectra was performed at different locations from which 3 major locations are reported here such as; (1) isocenter (source to center of PE sphere: 100 cm), (2) 50 cm from the isocenter (on patient treatment couch) and (3) maze-room junction. Figure 3 shows schematically the accelerator bunker with measurement locations 1, 2 and 3 in the treatment bunker.

As it was stated above, each of the 8 PE spheres has embedded a PE cube with 6 PCTD/^10^B detectors on sides. At each measurement location, the bare PCTD/^10^B and each of 8 PE spheres were exposed at a time to 5 Gy of 18 MV X-ray dose (at a depth of maximum dose) under a 10 × 10 cm^2^ X-ray field with respect to standard exposure conditions defined by TRS-398 (IAEA 2000)^36^.

### Photoneutron spectrum unfolding processes

#### Unfolding of PN energy spectrum

In situations where neutron energy spectra need to be determined, unfolding the neutron spectrum data is necessary. Neutron spectrometers generally do not directly determine neutron spectra. The basic experimental data usually come folded with the response of the used neutron detector. The energy spectra can be then estimated from the experimental data through a backward process called unfolding.

The relation between detector responses and neutron spectra can be written as Eq. (1):

1 $$A_{i} = \mathop \smallint \limits_{0}^{\infty } R_{i} \left( E \right) \cdot \varphi \left( E \right)dE\quad i = 1,2, \ldots ,n.$$

In Eq. (1), n is the number of detectors, Ai is the response of the i-th detector, Ri(E) is the i-th detector response as a function of neutron energy and φ(E) is neutron energy flux. Mathematically, the problem is the case of the Fredholm integral equation of the first kind. It does not have a unique solution, because a finite number of discrete measurements cannot define a continuous function. Therefore, the Eq. (1) can be rewritten in a matrix form as presented in Eq. (2):

2 $$A = R \cdot \varphi ,$$

where A is n × 1 vector of dosimeter response, R is n × m matrix of detector responses and φ is m × 1 vector of neutron energy flux. A number of mathematical methods have been applied to solve the problem such as least squares, iterative and Monte Carlo methods. In general, unfolding can be supposed as a mapping, from n dimensional space of detector response to m dimensional space of neutron energy flux.

The most complex aspect of neutron spectrometry with PE spheres is the unfolding process, due to the non-uniqueness of the solution. The unfolding of the PN spectrum determined by PE spheres is associated with several uncertainties from different sources such as those associated with the measurements, detector calibration and response functions^13,37,38^.

To unfold a neutron spectrum data obtained by a spectrometry method, some unfolding methods such as Monte Carlo^37,38^, parameterization and iterative procedures have been used. The time-consuming procedure and low-resolution spectrum of some methods applied^13,37,38^, motivated development of some complementary procedures such as maximum entropy Genetic Algorithms (GA) and Artificial Neural Networks (ANN)^13,39,40^. The ANNs are now being used in a wide variety of data processing applications. The ANN technique is suitable for unfolding the neutron spectra and has some advantages such as saving time and requiring few samples.

#### Artificial neural network (ANN) PN unfolding method

The unfolding process of the neutron energy spectrum using the ANN method is described. ANNs are mathematical models that are designed to think like the human brain^41,42^. Neural network models include interconnected groups of artificial neurons that imitate the function of neuron cells. Neural network models can learn from data using determined training rules^41,43^. Actually, neurons are the basic processing units of a network. A neuron takes weighted summation of multiple inputs to produce an output in accordance with a user specified transfer function. The weights for the inputs are known as “synaptic-weights”. A network can be regarded as a function that maps inputs to outputs. During the training process, a network adapts the synaptic-weights for a desired purpose^41–43^.

In the present study, to unfold the raw data obtained, an ANN-based code Multisphere Neutron Spectrometry Unfolding Code (MNSU+) for each MU was developed by us and used by which each set of PN fluence data at the center of each PE sphere was deconvolved into a 25-bin neutron energy spectrum. Practically, unlike other known and commonly used neutron unfolding methods, there is no need to form response matrix in ANN unfolding methods. Actually, in the other unfolding methods, the detection and evaluation were carried out in two steps; performing the measurement and unfolding of the detector responses. All of the unfolding methods need the detector response matrix for solving the problem and some of them need prior information about spectra. However, the neural networks are trained once, and then the trained neural network is used for unfolding, which takes a very short time without the need for a detector response matrix and any prior information^44–47^.

In the MNSU+, a simple linear ANN architecture without biases and hidden layers has been developed and trained for unfolding PN spectra. In each state, the PN fluence data at the center of each PE sphere and the generated PN energy spectrum are considered as input and output data of the ANN, respectively. In the training mode, the network is presented with a set of inputs corresponding to known outputs. During the training it learns the rules that produce the outputs from the inputs and the weights are adjusted to present the closest outputs to the real outputs. In this study, the training data are sets of neuron spectra defined from thermal to 630 MeV in sixty energy groups and the corresponding responses of Bonner spheres taken from IAEA's Compendium of neutron spectra and detector responses for radiation protection purposes (IAEA Technical Report Series No.318)^48^, a set of response functions of monoenergetic neutrons at the center of each PE sphere provided by Standard Dosimetry Laboratory of the National Radiation Protection Department (Tehran, Iran) and determined at the center of each PE sphere and also calculated by simulation using MCNP 4C Monte Carlo code. The training method is the resilient backpropagation algorithm. An energy spectra measurement system was also set up for the validation of the unfolding capability by using a ^252^Cf neutron source. The resultant unfolded ^252^Cf source spectrum is in agreement with the results provided in the literature for neutron spectrometry^11,20–22,37,38,49^.

## Experimental findings and results

**Figure 4:**
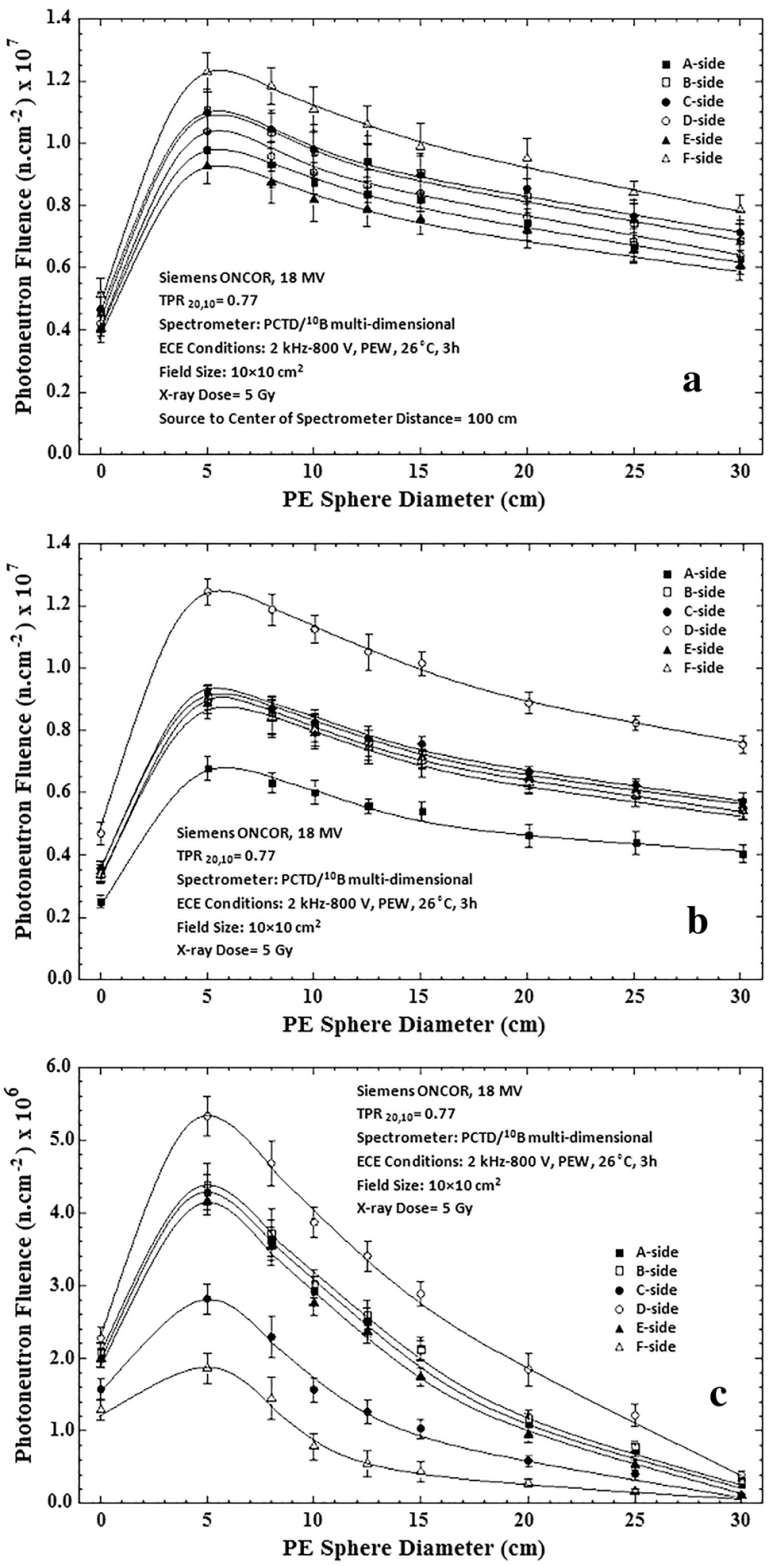
Photoneutron fluence versus diameter of the PE spheres for all six side PCTD/^10^B detectors as determined at 3 spectrometry locations in the radiotherapy bunker; (**a**) isocenter, (**b**) 50 cm from isocenter, and (**c**) maze-room junction for 5 Gy dose of 18 MV X-rays in a 10 × 10 cm^2^ field of the medical accelerator.

Photoneutron spectrometry of 3 major locations in the radiotherapy bunker of the medical accelerator was carried out in particular at the isocenter with different field sizes using the multi-directional neutron spectrometry system developed in this study. One bare PE cube with 6 side PCTD/^10^B detectors and 8 PE spheres embedding PE cubes at their centers have been exposed one at a time at each location under the same 18 MV X-ray dose of 5 Gy. Each measurement location simply leads to 54 data points based on which the PN fluences have been determined for all the 6 directions. Each side detector of a PE cube, used either as bare or as embedded in a sphere, at each measurement location provides spectrum data different from those of other sides of the PE cube. This is due to scattering of fast PNs from the surrounding media caused by different geometry conditions in locations in the radiotherapy bunker. Such variations can be well demonstrated by plotting the 54 data points taken at each of the 3 measurement locations in the radiotherapy bunker as functions of the PE sphere diameter. Figure 4a–c demonstrates the PN fluence versus PE sphere diameter for each PCTD/^10^B detector on each side of the PE cube at 3 spectrometry locations in the bunker at: (a) isocenter, (b) 50 cm from the isocenter, and (c) maze-room junction for 5 Gy dose of 18 MV X-rays in a 10 × 10 cm^2^ field of the medical accelerator.

By direct analysis of the 3 sets of responses of Fig. 4a–c, some conclusions follow:

1. In order to better analyze the PN fluence versus PE sphere diameter responses, one has to understand what each PCTD/^10^B on a PE cube side detects. In general, each side PCTD/^10^B detects neutrons from all directions, but preferably from the direction facing the side. For example, the PCTD/^10^B on side A at the isocenter primarily detects neutrons coming from the direct beam and those coming from scattered PNs of other directions. Therefore, since thermal neutron fluence directly from the beam is relatively low, the full thermal neutron response of the side A is below those of the sides B and D which receive more thermalized neutrons from the surrounding media. Such explanations can be extended to also other side detector responses.
2. The data of the bare PE cube with detectors on sides have the lowest values in all 3 sets of responses. In particular, at the isocenter, they have more or less the same values close to each other. These values are more diverted at other two PN spectrometry locations.
3. In all 3 sets of responses, 5 cm diameter sphere data show maximum thermal neutron fluences or peaks for each of 6 sides.
4. The responses of the 30 cm diameter PE sphere at the maze-room junction is about 10 times lower than relevant data at the isocenter and at 50 cm from the isocenter due to the PN spectrum being well softened at that spectrometry location.
5. The responses at the isocenter are more structured relative to each other with the same trend while those of the other two spectrometry locations are more diverted.

The PN spectra at the three stated locations were all determined with X-ray dose of 5 Gy. However, such data are reported in Table 1 as PN fluence per MU for six PCTD/^10^B detectors on sides A, B, C, D, E, and F of PE cubes embedded at the centers of all 8 PE spheres exposed at the isocenter based on which the PN spectra and their mean spectrum have been determined.

**Table 1:** PN spectrum data with mean values of PN fluence per MU of 6 PCTD/^10^B detectors on sides A, B, C, D, E, and F of PE cubes either used as bare or embedded at the centers of all 8 PE spheres at the isocenter (on the treatment couch) exposed to 5 Gy of18 MV X-ray dose in a 10 × 10 cm^2^ beam size of the beam of the medical accelerator.

Energy (MeV)	Photoneutron Fluence per MU (n cm^−2^ MU^−1^)						
	A	B	C	D	E	F	Mean
1.03E−09	1.02E+04	1.03E+04	9.05E+03	1.10E+04	8.36E+03	1.01E+04	9.84E+03
5.78E−09	6.57E+04	6.57E+04	2.70E+04	7.19E+04	5.13E+04	6.50E+04	6.29E+04
1.87E−08	3.14E+05	3.14E+05	2.75E+05	3.45E+05	2.49E+05	3.12E+05	3.02E+05
3.28E−08	6.96E+05	6.96E+05	6.04E+05	7.02E+05	5.44E+05	6.89E+05	6.66E+05
5.78E−08	6.93E+05	6.93E+05	6.46E+05	7.62E+05	5.51E+05	6.88E+05	6.66E+05
1.03E−07	3.41E+05	3.41E+05	2.98E+05	3.75E+05	2.71E+05	3.38E+05	3.28E+05
3.28E−07	2.97E+05	2.97E+05	2.90E+05	3.26E+05	2.36E+05	2.95E+05	2.86E+05
1.03E−06	1.90E+05	1.90E+05	1.64E+05	1.88E+05	1.49E+05	1.87E+05	1.82E+05
3.28E−06	1.70E+05	1.70E+05	1.47E + 05	1.86E+05	1.31E+05	1.67E+05	1.62E+05
1.03E−05	1.67E+05	1.67E+05	1.44E+05	1.81E+05	1.31E+05	1.64E+05	1.60E+05
3.28E−05	1.69E+05	1.69E+05	1.47E+05	1.86E+05	1.34E+05	1.67E+05	1.62E+05
1.03E−04	1.65E+05	1.65E+05	1.44E+05	1.81E+05	1.31E+05	1.63E+05	1.58E+05
3.28E−04	1.66E+05	1.66E+05	1.44E+05	1.82E+05	1.32E+05	1.64E+05	1.59E+05
1.03E−03	1.61E+05	1.61E+05	1.40E+05	1.85E+05	1.30E+05	1.58E+05	1.55E+05
3.28E−03	1.97E+05	1.97E+05	1.51E+05	2.54E+05	1.31E+05	1.93E+05	1.89E+05
1.03E−02	2.06E+05	2.06E+05	1.79E+05	2.64E+05	1.66E+05	2.02E+05	1.97E+05
3.28E−02	4.00E+05	4.00E+05	3.52E+05	4.35E+05	3.24E+05	3.93E+05	3.84E+05
5.78E−02	9.79E+05	9.79E+05	7.62E+05	1.07E+06	7.93E+05	9.62E+05	9.42E+05
1.03E−01	1.66E+06	1.66E+06	1.36E+06	1.81E+06	1.65E+06	1.63E+06	1.60E+06
1.87E−01	2.27E+06	2.27E+06	2.02E+06	2.41E+06	1.89E+06	2.27E+06	2.19E+06
3.28E−01	1.36E+06	1.36E+06	1.21E+06	1.47E+06	1.29E+06	1.36E+06	1.31E+06
5.78E−01	1.09E+06	1.09E+06	9.74E+05	1.18E+06	8.77E+05	1.09E+06	1.05E+06
1.03E+00	5.52E+05	5.52E+05	4.89E+05	5.97E+05	4.35E+05	5.45E+05	5.29E+05
1.87E+00	5.49E+04	5.49E+04	1.49E+05	2.09E+05	1.33E+04	5.43E+04	5.27E+04
1.03E+01	2.89E+03	2.89E+03	2.56E+03	3.12E+03	2.28E+03	2.85E+03	2.77E+03

**Figure 5:**
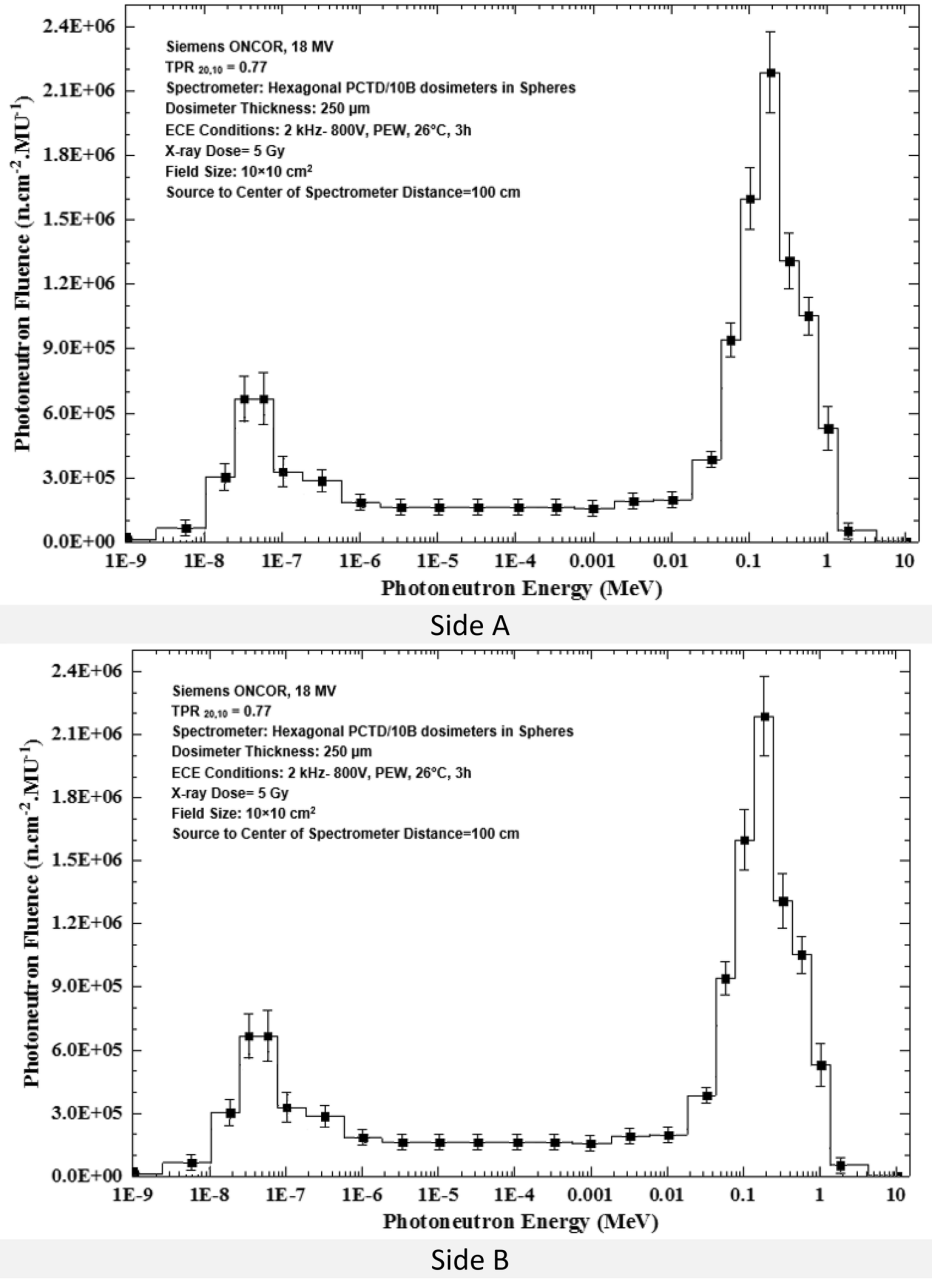

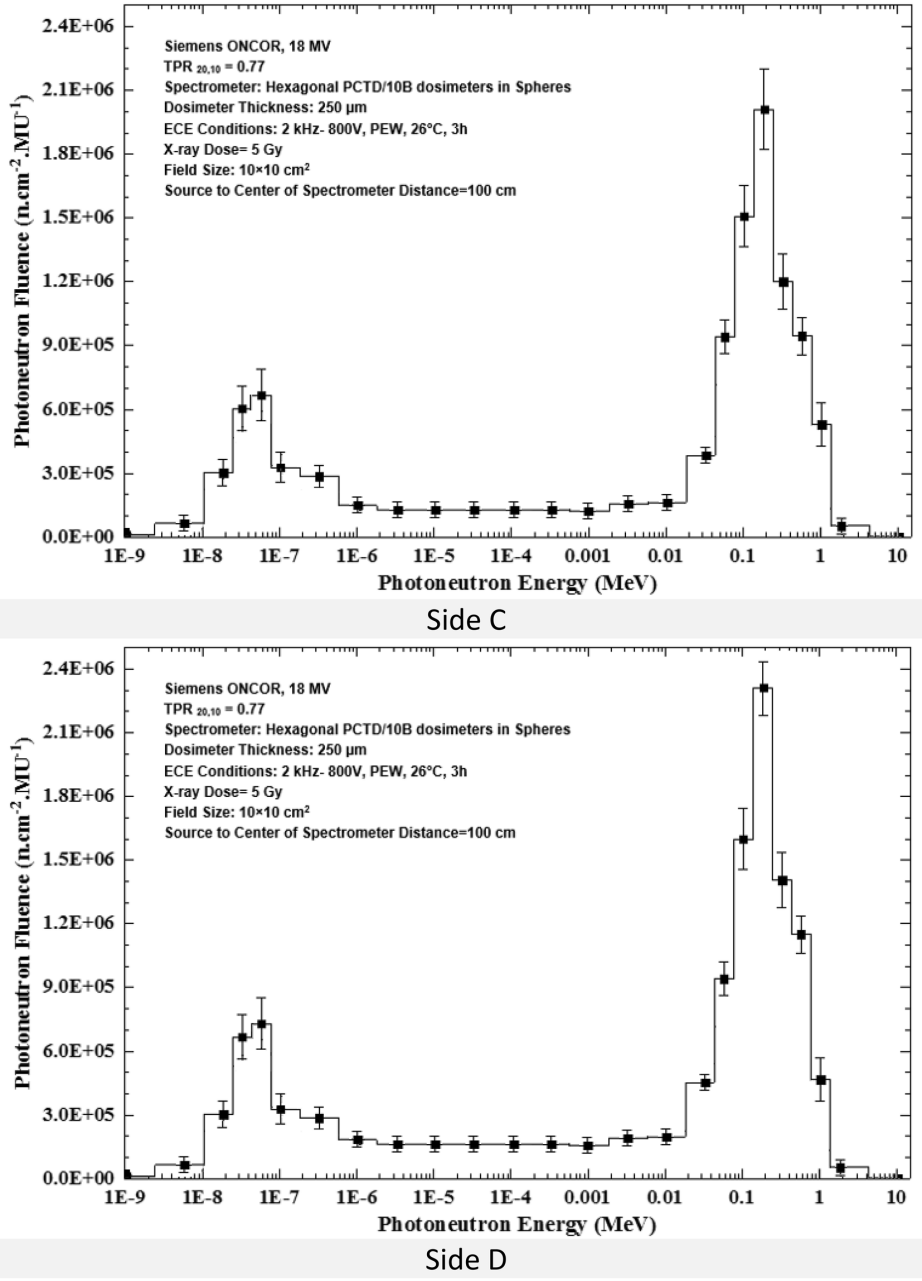

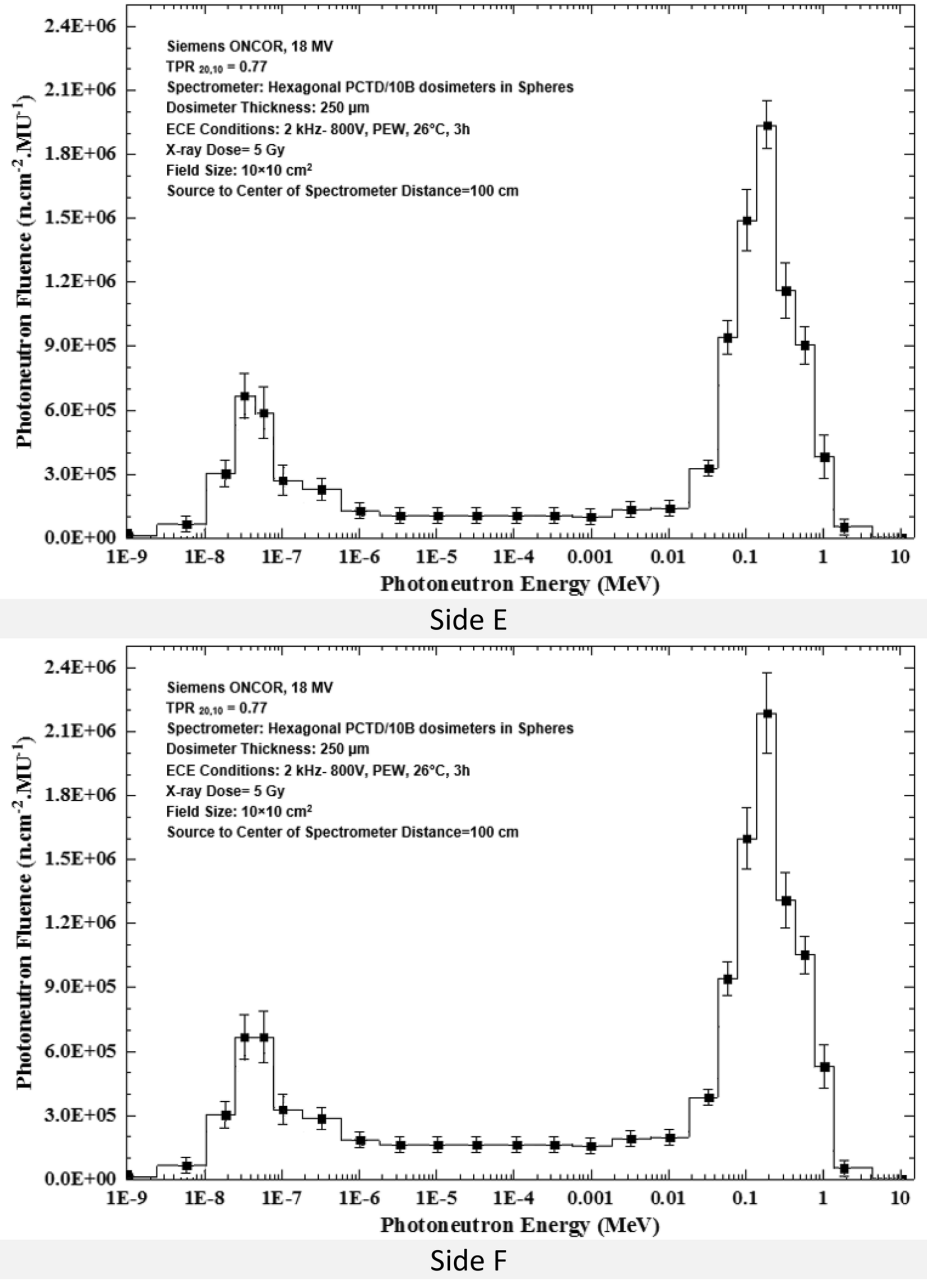
(**a–f**) PN spectra determined by the 6 PCTD/^10^B detectors fixed on 6 sides A, B, C, D, E, and F of PE cubes (one bare and 8 embedded in 8 PE spheres) exposed to PNs at the isocenter to 5 Gy 18 MV X-ray dose in the 10 × 10 cm^2^ field.

Applying the basic data given in Table 1, the PN spectra determined by 6 PCTD/^10^B detectors on 6 sides of the 9 PE cubes (one bare and 8 embedded in PE spheres) as exposed at the isocenter to 5 Gy 18 MV X-ray dose in a 10 × 10 cm^2^ field are shown in Fig. 5 (a,b,c,d,e,f).

By direct analysis of the basic data the PN spectra given in Table 1 and in Fig. 5a–f, some conclusions can be drawn;

1. Regardless of the side of the PE cube, the general shape of the PN spectra of the 6 side detectors are close to each other and well demonstrates two peaks; one thermal PN peak with an epithermal and intermediate energy tail followed by another peak for fast PNs within 0.05 to 1.0 MeV^2,8,31,39,40,50,51^.
2. Thermal PNs have small contribution, while fast PNs have the largest influence on the total PN dose equivalent. Previous studies showed that about 90% of PN dose equivalent was contributed by fast PNs with energy above 0.5 MeV^2,5,7,8,13,20,32,33,40,51^.
3. The PN fluences determined by detectors on 6 sides of the PE cube are quite different. For example, the PN fluences on the sides C and F are in general lower than the others almost at all energies.

**Figure 6:**
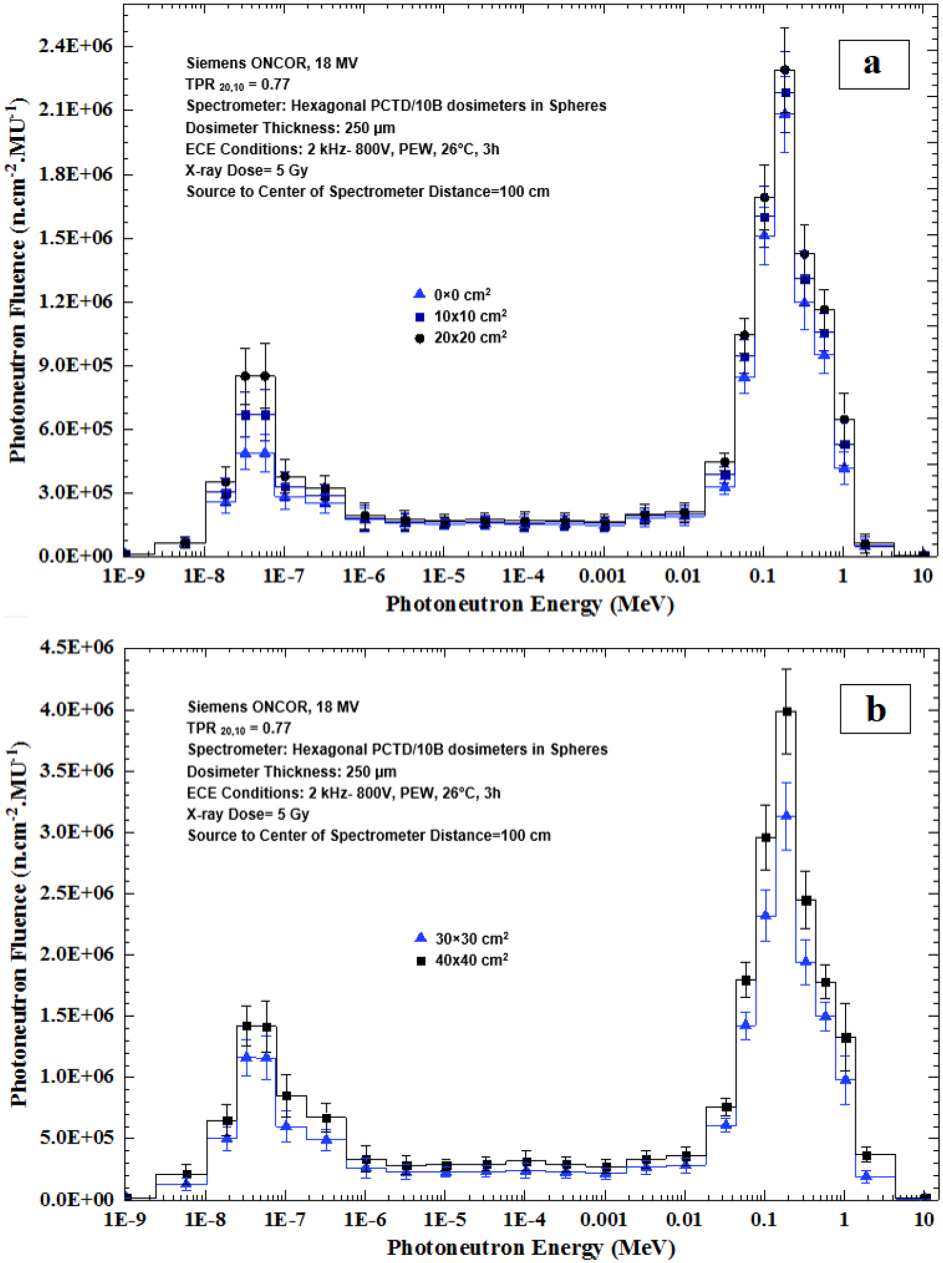
PN spectra of 5 field sizes; (**a**) 0 × 0, 10 × 10 and 20 × 20 cm^2^ and (**b**) 30 × 30 and 40 × 40 cm^2^, by the PE sphere neutron spectrometry system developed as exposed at the isocenter to 5 Gy 18 MV X-ray dose.

It has been recently demonstrated by us that fast, epithermal and thermal PN dose equivalents increase linearly as field size increases for field sizes 10 × 10, 20 × 20, 30 × 30 and 40 × 40 cm^2^ studied^33,39,50^, as also discussed by others^13,52,53^. This can be due to less attenuation of the PNs by the accelerator head material components as the field size increases, and less scattering from the target, beam flattening filter and primary collimator. This effect of course can cause the neutron spectrum to differ from one field size to another. Therefore, in order to further verify this concept by PN spectrometry, the PN spectra of 5 field sizes 0 × 0, 10 × 10, 20 × 20, 30 × 30 and 40 × 40 cm^2^ were determined at the isocenter. Figure 6a,b shows PN spectra of 5 field sizes; (a) 0 × 0, 10 × 10 and 20 × 20 cm^2^, and (b) 30 × 30 and 40 × 40 cm^2^ as exposed at the isocenter to 5 Gy 18 MV X-ray dose.

**Figure 7:**
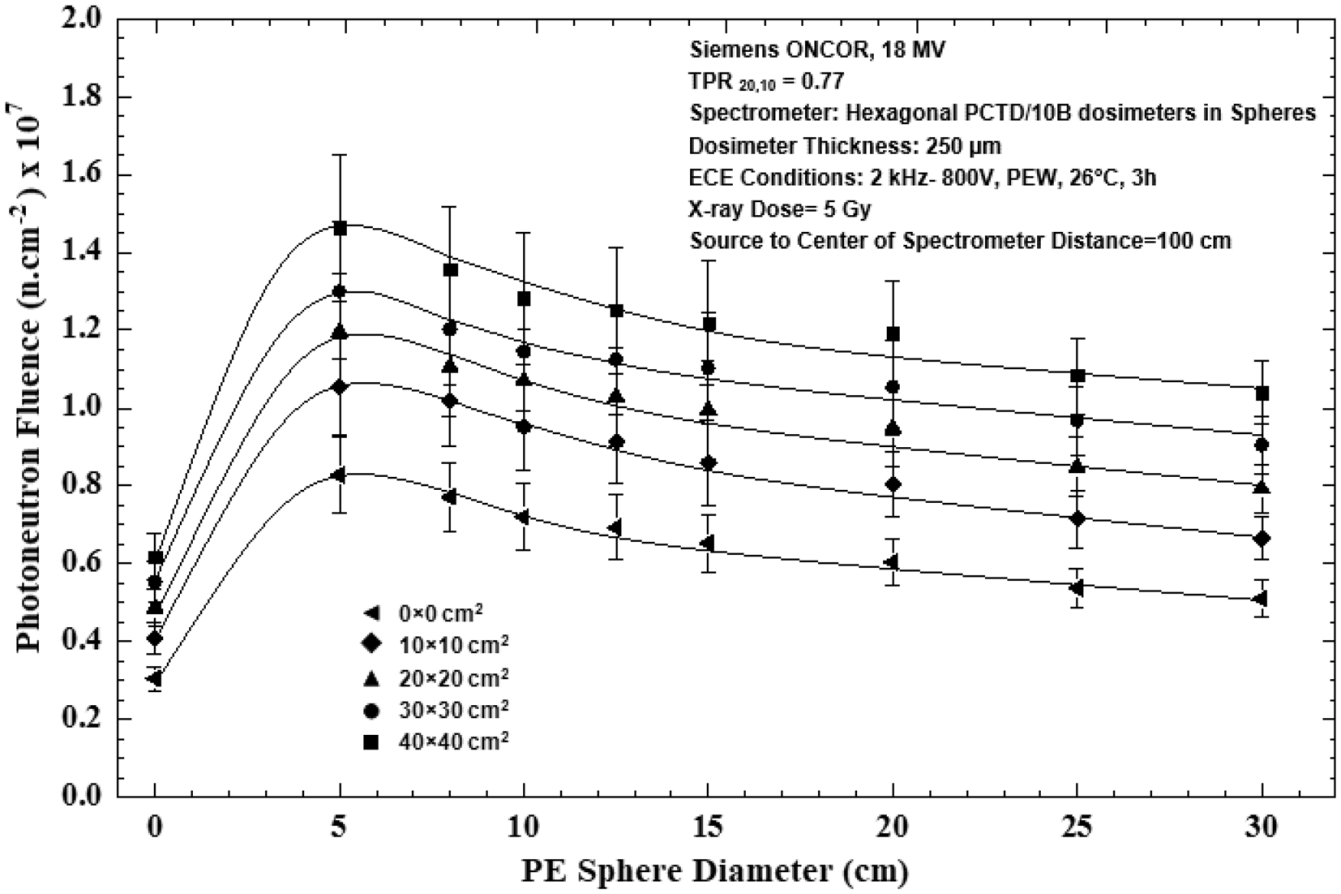
Photoneutron fluence versus PE sphere diameter for 0 × 0, 10 × 10, 20 × 20, 30 × 30 and 40 × 40 cm^2^ field sizes at the isocenter exposed to 5 Gy 18 MV X-ray dose.

The PN spectra in Fig. 6a,b for the 5 field sizes well demonstrate an increase in the PN fluence and are hardened in neutron energy as the field size has increased. However, in order to better demonstrate the effects of field size on the PN fluence, the PN fluence as functions of diameter of the PE spheres for 5 different field sizes 0 × 0, 10 × 10, 20 × 20, 30 × 30 and 40 × 40 cm^2^ are shown in Fig. 7.

**Figure 8:**
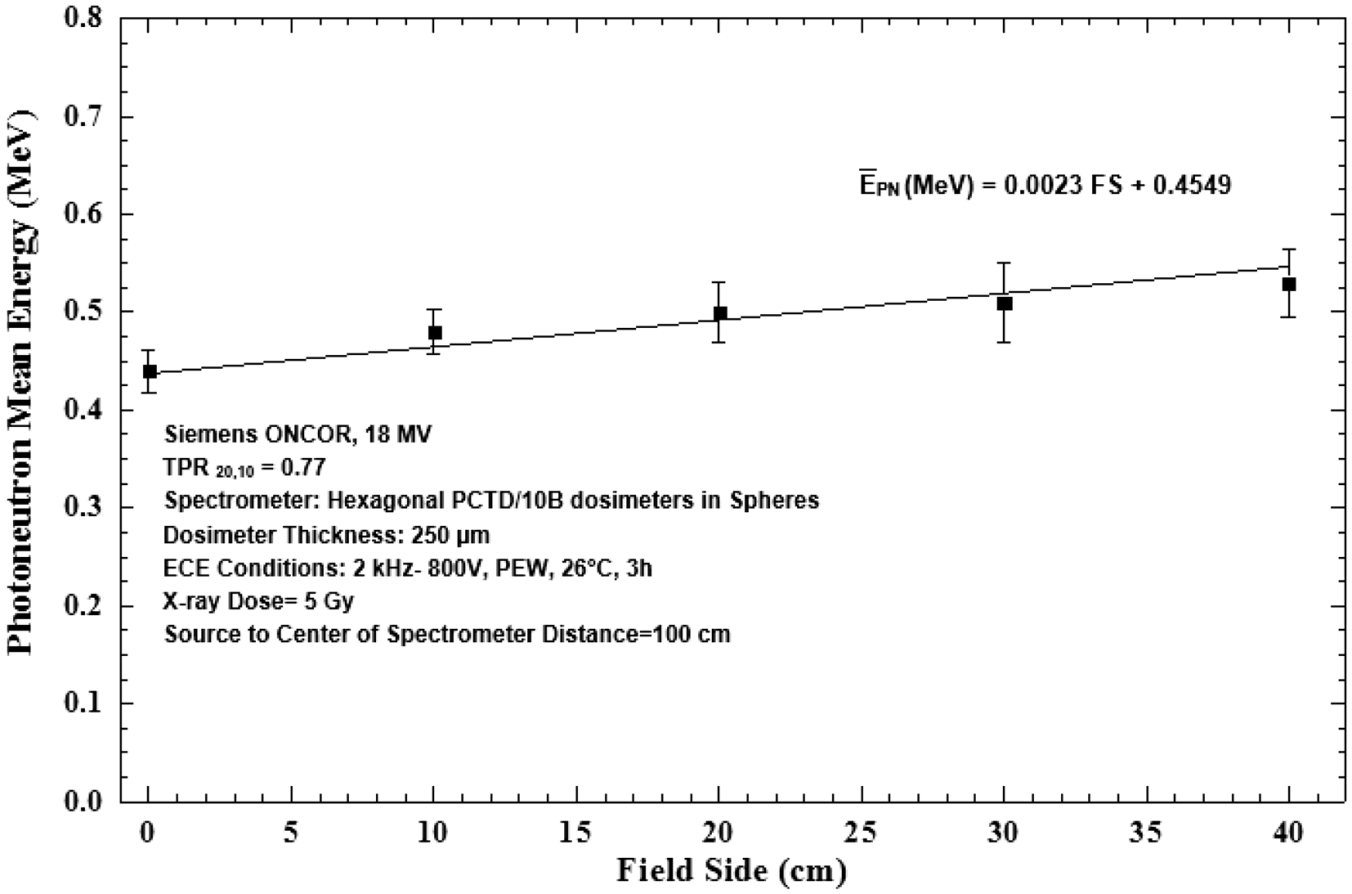
Photoneutron mean energy versus field side for field sizes 0 × 0, 10 × 10, 20 × 20, 30 × 30 and 40 × 40 cm^2^ at the isocenter of 18 MV X-ray beams of the medical accelerator.

The PN fluence versus PE sphere diameter responses of Fig. 7 well demonstrate that as the field size has increased, the PN fluence at all energies has increased. This observation indicates also that the mean energy of PNs in general has increased as the field size has increased, as shown in Fig. 8. The PN mean energy versus field size response as shown in Fig. 8 is linear and the mean PN energies obtained are 0.44 ± 0.01, 0.48 ± 0.02, 0.50 ± 0.03, 0.51 ± 0.04, 0.53 ± 0.03 MeV respectively for field sizes 0 × 0, 10 × 10, 20 × 20, 30 × 30 and 40 × 40 cm^2^.

By direct analysis of the 5 PN energy spectra of 5 field sizes, as shown in Figs. 6a,b, 7 and 8, some conclusions follow;

1. All five PN energy spectra of 5 field sizes of Fig. 6a,b demonstrate a common spectrum trend with two peaks; one thermal PN peak followed by a tail of epithermal and intermediate energy PNs before reaching a fast PN peak, as has also been observed by others^2,13^.
2. As field size has increased, the PN fluence levels at all energies have increased. In particular, the heights of the thermal and fast PN peaks have increased as the field size has increased; i.e. the peak heights are the lowest for the 0 × 0 cm^2^ field and the highest for 40 × 40 cm^2^ field sizes.
3. The mean PN energy has also increased as the field size has increased (Fig. 8).

The above conclusions are all in support of the findings that the fast, epithermal and thermal PN dose equivalents increase linearly as field size increases^13,31,33,50,52,53^.

**Figure 9:**
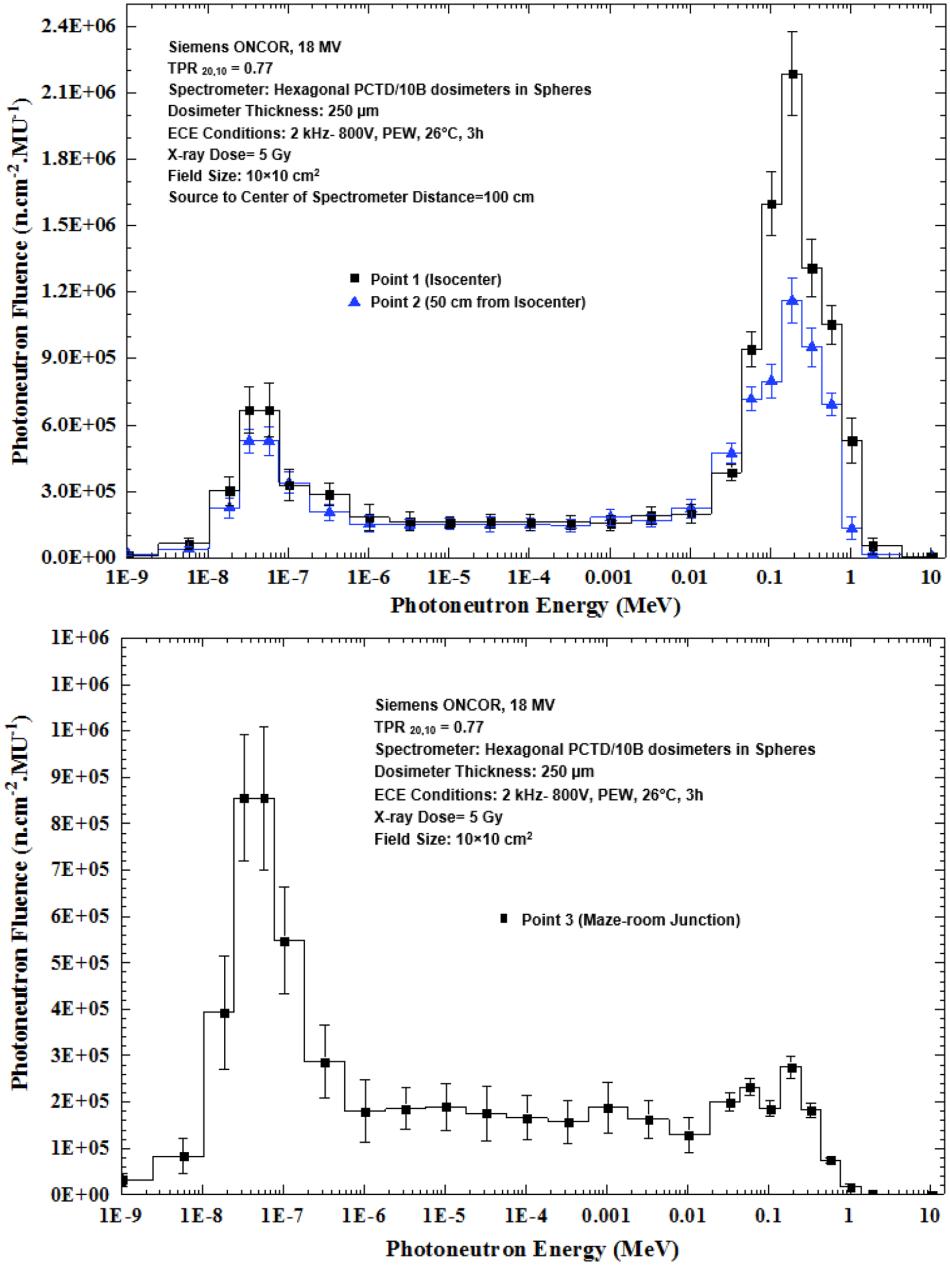
The PN spectra determined at 3 locations in the accelerator bunker; the isocenter, 50 cm from the isocenter, and at the maze-room junction in a 10 × 10 cm^2^ field for 5 Gy 18 MV X-rays dose.

As shown in Fig. 6a,b, the PN spectra obtained at the isocenter have a low-height thermal fluence peak and a sharp large peak due to fast neutrons. While the fast neutron peak can be more or less related to the main beam, the thermal PN peak is partly from the beam and partly from fast PNs thermalized and reduced in energy through elastic scattering with atomic constituents of the material in the accelerator head, and surrounding media. Such elastic scattering interactions in fact make the PN spectra in a high energy X-ray therapy bunker vary from location to location. Therefore, in order to further demonstrate such variations, PN spectra of different locations in the radiotherapy bunker at the three locations namely the isocenter, 50 cm from the isocenter and at the maze-room junction are compared, as shown in Fig. 9. The X-ray dose was 5 Gy in a 10 × 10 cm^2^ field size of the 18 MV X-ray beam.

By analyzing the 3 PN spectra of Fig. 9 at the 3 stated locations comparatively, some conclusions follow:

1. The PN spectrum has been strongly softened when distance from the isocenter to the other two locations has increased; slightly at 50 cm from the isocenter and strongly at the maze-room junction.
2. The thermal PN peak height of each PN spectrum strongly increases and the fast PN peak height strongly decreases as distance has increased from the isocenter to the maze-room junction. This is due to softening of the fast PNs scattered by the surrounding media.
3. The neutron spectrometry system developed was demonstrated being highly sensitive to detect any small changes occurred in the PN fluence and energy at different spectrometry locations in the radiotherapy bunker. In fact, the PN spectra at different locations of the maze which have very low PN fluences with relatively softer spectra have been determined which demonstrate the stated claim and will be reported elsewhere.

The general shape of the PN spectra are in good agreement with those of other studies using different PE sphere spectrometry methods with other central thermal PN detectors such as activation foils^31,40^, superheated drop detectors^50^, thermoluminescence detectors^13,39,40^ and CR-39^51^, as well as simulation of the medical accelerator head and treatment conditions by using simulation codes^2,49,50^.

## Discussions

The rationale for developing the passive multi-directional multi-detector spherical neutron spectrometry system in this study is its high sensitivity to neutrons, insensitivity to low-LET radiation (x, γ and β) and non-ionizing radiation, negligible inherent geometric directional dependence, no post-exposure fading, simple and small detectors, easily home-made, no need to sophisticated detectors/instruments, reproducible in any developing or developed laboratories, low cost, no need to cable connection, scalable and applicable to other applications from small to mega-size detectors, simple record keeping of processed detectors, ease of manpower training, and in particular capable in determining directional and mean neutron spectra of a mixed neutron-gamma field. The neutron spectrometry system developed fulfils these requirements in particular the PE spheres are isotropic in response and have negligible inherent geometric directional dependency compared to other PE moderator geometries. The insensitivity of PCTDs to low-LET radiation has been proved in our pioneering studies by exposing them to a γ dose of 10^2^ Gy from a ^137^Cs source^32^.

Albeit the unique characteristics of the passive neutron spectrometry system developed, it has some disadvantages such as lack of online provision of data (like any other passive systems), need to ECE processing for PCTDs by applying high voltage power supplies at a certain frequency (what some laboratories might have hesitations to apply; but if CR-39 is used, chemical etching can be used), and also manual track counting when an automatic track counting system is not applied. In fact, if the system is well established by a developing or developed laboratory or a service provider, services may be provided for national, regional, and possibly international scientific collaborations or for provision of commercial services to other laboratories at a reasonable cost. This approach can be simply made by only mailing PE cube (s) with 6 PCTD/^10^B thermal neutron detectors on sides; being passive system in fact is highly advantageous. Only one or two cubes with some additional PCTDs are enough since only one PE sphere is exposed at a time and only the PCTDs can be changed and the same cube can be reused. The service providing laboratory or company then receives the exposed PCTDs, processes them, count the tracks either manually or by a processing system, unfolds the data and reports the results; either as scientific collaborations or by payment. The laboratory receiving services can only prepare a set of PE spheres in order to obtain the full neutron spectrum, or by any other arrangements needed^31^. Another added value of this passive system is insensitivity to low-LET radiation so that it can be used in exotic or extreme mixed field radiation applications such as on board of space shuttle and international space stations as well as for other cosmic ray studies, if online information can be tolerated. In particular, the system as being passive can be exposed to cosmic neutrons in an environment for a long time on the ground or at a desired elevation. By this approach, the cosmic neutron spectrum can be determined with very high accuracy.

The multi-directional neutron spectrometry system introduced in this study provides the capability for capturing PNs directionally as demonstrated in an exotic or extreme application having low fluence neutrons in the presence of 5 Gy of 18 MV X-rays in a medical accelerator. The PN spectrometry data reported above demonstrated well-resolved neutron spectra even at situations where the neutron spectra were very close to each other. The PN spectra all showed well-resolved thermal PN peaks along with epithermal and intermediate energy tail reaching a fast PN peak the heights of which depend on many factors discussed. The general mean PN spectra trend is in good agreement with those of other studies using different PN spectrometry methods and of course with differences as regards to X-ray energy, type of head material, type of detector, radiotherapy bunker design and construction material, spectrometry location, etc.^13,31,39,40,50,52,53^.

## Conclusions

A passive multi-directional multi-detector spherical neutron spectrometry system, with some unique characteristics, as discussed above, was designed, home-made and applied to an exotic PN spectrometry in a medical accelerator bunker. The system used 6 PCTD/^10^B detectors on 6 sides of a PE cube used as bare or embedded at the center of 8 PE spheres of different sizes in order to determine directional neutron spectra and mean spectra of locations of concern in a Siemens ONCOR medical linear accelerator bunker. The neutron spectrometry system was successful in obtaining:

- Well-resolved PN fluence responses as functions of PE sphere diameters for 6 different directions at the isocenter, at 50 cm from the isocenter and at the maze-room junction pf the accelerator bunker (Fig. 4a–c),
- Six well-resolved directional and mean PN spectra of a 10 × 10 cm^2^ 18 MV X-ray field at the isocenter as well as mean PN spectra of other field sizes and locations (Fig. 5a–f),
- Five well-resolved mean spectra at the isocenter for 5 field sizes 0 × 0, 10 × 10, 20 × 20, 30 × 30 and 40 × 40 cm^2^ distinctly distinguished from each other (Fig. 6), and

The neutron spectrometry system introduced and used in such an exotic PN spectrometry application has well demonstrated that the system can be easily and successfully applied for neutron spectrometry in other areas in medicine, health, environment, science and technology in any developed or developing laboratories.

## References

[1] Sohrabi M, Hakimi A (2020). Novel ‘photoneutron volume dose equivalent’ hypothesis and methodology for second primary cancer risk estimation in high-energy X-ray medical accelerators. Radiat. Prot. Dosim..

[2] Maglieri R, Licea A, Evans M, Seuntjens J, Kildea J (2015). Measuring neutron spectra in radiotherapy using the nested neutron spectrometer. Med. Phys..

[3] Hu J, Liu J, Zhang Z, Chen L, Guo Y, He S, Xu M, Zhou L, Yao Z, Yuan X, Zhang Q (2018). Recoil-proton track imaging as a new way for neutron spectrometry measurements. Sci. Rep..

[4] Maspero M, Berra A, Conti V, Giannini G, Ostinelli A, Prest M, Vallazza E (2015). A real time scintillating fiber time of flight spectrometer for linac photoproduced neutrons. Nucl. Instrum. Method A..

[5] McGinley, P. & Sohrabi, M. Neutron contamination in the primary beam. In *Proceedings of Conference on Neutrons from Electron Medical Accelerators: held at the National Bureau of Standards, Gaithersburg, Maryland*, April 9–10 (1979).

[6] Guardiola C, Gómez F, Fleta C, Rodríguez J, Quirion D, Pellegrini G, Lousa A, Martínez-de-Olcoz L, Pombar M, Lozano M (2013). Neutron measurements with ultra-thin 3D silicon sensors in a radiotherapy treatment room using a Siemens PRIMUS linac. Phys. Med. Biol..

[7] Howell RM, Hertel NE, Wang Z, Hutchinson J, Fullerton GD (2006). Calculation of effective dose from measurements of secondary neutron spectra and scattered photon dose from dynamic MLC IMRT for 6MV, 15MV, and 18MV beam energies. Med. Phys..

[8] Esposito A, Bedogni R, Lembo L, Morelli M (2008). Determination of the neutron spectra around an 18MV medical LINAC with a passive Bonner sphere spectrometer based on gold foils and TLD pairs. Radiat. Meas..

[9] McCall RC, Jenkins TM, Shore RA (1979). Transport of accelerator produced neutrons in a concrete room. IEEE. Trans. Nucl. Sci..

[10] McGinley, P. & Sohrabi, M. Dose levels due to neutrons in the vicinity of high energy medical accelerators. In *Proceedings 9th Midyear Topical Symp, Health Physics Society. Denver, Colorado*, 9–12 Feb. 468–474 (1976). 10.1118/1.594256 826776

[11] Thomas DJ, Alevra AV (2002). Bonner sphere spectrometers—A critical review. Nucl. Instrum. Methods Phys. Res. B..

[12] Gracanin V, Guatelli S, Prokopovich D, Rosenfeld AB, Berry A (2017). Development of a Geant4 application to characterise a prototype neutron detector based on three orthogonal ^3^He tubes inside an HDPE sphere. Phys. Med..

[13] Kralik M, Turek K, Vondráček V (2008). Spectra of photoneutrons produced by high-energy X-ray radiotherapy linacs. Radiat. Prot. Dosim..

[14] Caresana M, Agosteo S, Campi F, Ferrarini M, Porta A, Silari M (2007). Sensitivity study of CR39 track detector in an extended range Bonner sphere spectrometer. Radiat. Prot. Dosim..

[15] Caresana M, Ferrarini M, Pola A, Agosteo S, Campi F, Porta A (2010). Study of a radiator degrader CR39 based neutron spectrometer. Nucl. Instrum. Methods. Phys. Res. B..

[16] Paul S, Tripathy SP, Sahoo GS, Bandyopadhyay T, Sarkar PK (2013). Measurement of fast neutron spectrum using CR-39 detectors and a new image analysis program (autoTRAK_n). Nucl. Instrum. Methods Phys. Res. B..

[17] Ongaro C, Zanini A, Nastasi U, Ródenas J, Ottaviano G, Manfredotti C (2000). Analysis of photoneutron spectra produced in medical accelerators. Phys. Med. Biol..

[18] d'Errico F, Nath R, Tana L, Curzio G, Alberts WG (1998). In-phantom dosimetry and spectrometry of photoneutrons from an 18 MV linear accelerator. Med. Phys..

[19] d'Errico F, Giusti V, Reginatto M, Wiegel B (2004). A telescope-design directional neutron spectrometer. Radiat. Prot. Dosim..

[20] Barquero R, Edwards TM, Iñiguez MP, Vega-Carrillo HR (2005). Monte Carlo simulation estimates of neutron doses to critical organs of a patient undergoing 18MV X-ray LINAC-based radiotherapy. Med. Phys..

[21] Gómez-Ros JM, Bedogni R, Moraleda M, Delgado A, Romero A, Esposito A (2010). A multi-detector neutron spectrometer with nearly isotropic response for environmental and workplace monitoring. Nucl. Instrum. Methods Phys. Res. B..

[22] Bedogni R, Bortot D, Buonomo B, Esposito A, Gómez-Ros JM, Introini MV, Romero AM (2016). A single-exposure, multidetector neutron spectrometer for workplace monitoring. Radiat. Prot. Dosim..

[23] International Commission on Radiological Protection, Recommendations of the International Commission on Radiological Protection. ICRP Publication 103. Ann ICRP 37 (2007). 10.1016/j.icrp.2007.10.003 18082557

[24] Bedogni R, Gómez-Ros JM, Lega A, Menzio L, Moraleda M, Pola A, Pietropaolo A, Ferrante Vero L (2020). Design of an ultra-sensitive single-moderator directional neutron spectrometer based on 3He detectors. Nucl. Instrum. Method A..

[25] Sohrabi M (1974). The amplification of recoil particle tracks in polymers and its application in fast neutron personnel dosimetry. Health. Phys..

[26] Sohrabi M (1979). A new dual response albedo neutron personnel dosimeter. Nucl. Instrum. Method.

[27] Sohrabi M, Morgan KZ (1978). A new polycarbonate fast neutron personnel dosimeter. Am. Ind. Hyg. Assoc. J..

[28] Sohrabi M, Katouzi M (1991). Design characteristics of a three-component AEOI Neutriran albedo neutron personnel dosimeter. Int. J. Radic. Appl. Instrum. D..

[29] Sohrabi M, Soltani Z (2021). A new application of Sohrabi Albedo Neutron Dosimeters around a plasma focus device. Health. Phys..

[30] Sohrabi M (2017). Novel single-cell mega-size chambers for electrochemical etching of panorama position-sensitive polycarbonate ion image detectors. Rev. Sci. Instrum..

[31] Sohrabi M, Hakimi A (2017). Novel 6 MV X-ray photoneutron detection and dosimetry of medical accelerators. Phys. Med..

[32] Sohrabi M, Morgan KZ (1979). Neutron dosimetry in high energy X-ray beams of medical accelerators. Phys. Med. Biol..

[33] Hakimi A, Sohrabi M, Mahdavi SR (2017). Effects of field size and depth on photoneutron dose equivalent distributions in an 18 MV X-ray medical accelerator. Radiat. Prot. Dosim..

[34] Sohrabi M, Hakimi A, Soltani Z (2015). Background track density reduction of 50-Hz–HV ECE-processed thick polycarbonate detectors to improve lower detection limit. Radiat. Prot. Dosim..

[35] Sohrabi M, Soltani Z, Hakimi A (2016). Combined effects of frequency and layer removal on background track characteristics of ECE polycarbonate detectors. Radiat. Phys. Chem..

[36] International Atomic Energy Agency. Absorbed dose determination in external beam radiotherapy: an international code of practice for dosimetry based on standards of absorbed dose to water. Technical Report Series No. 398. Vienna: IAEA (2000).

[37] Sanna R, O’Brien K (1971). Monte-Carlo unfolding of neutron spectra. Nucl. Instrum. Method A..

[38] Lindemann L, Zech G (1995). Unfolding by weighting Monte Carlo events. Nucl. Instrum. Method Phys. Res. A..

[39] Sohrabi M, Hakimi A, Mahdavi SR (2016). A novel position-sensitive mega-size dosimeter for photoneutrons in high-energy X-ray medical accelerators. Phys. Med..

[40] Sohrabi M, Hakimi A (2018). Fast, epithermal and thermal photoneutron dosimetry in air and in tissue equivalent phantom for a high-energy X-ray medical accelerator. Z. Med. Phys..

[41] Haykin, S. *Multilayer Perceptrons. Neural networks: A comprehensive fundationed*, vol. 2, 156–255 (Prentice-Hall, New Jersey, 1999).

[42] Hosseini AM (2016). Neutron spectrum unfolding using artificial neural network and modified least square method. Radiat. Phys. Chem..

[43] Agatonovic-Kustrin S, Beresford R (2000). Basin concepts of artificial neural network (ANN) modeling and its application in pharmaceutical research. J. Pharm. Biomed. Anal..

[44] McElroy, W.N., Berg, S., Crockett, T. & Hawkins R.G. *A computer-automated iterative method for neural flux spectra determination by foil activation*. Report AFWL-TR-64-41 (U.S. Air Force Weapons Laboratory) (1976).

[45] Cordes E, Fahrenbacher G, Schutz R, Sprunk M, Hahn K, Hofman R, Biersack JP, Wahl W (1998). An approach of unfold the response of multi-element system using an artificial neural network. IEEE Trans. Nucl. Sci..

[46] Braga CC, Dias MS (2002). Application of neural network for unfolding neutron spectra measured by means of Bonner spheres. Nucl. Instrum. Methods. A..

[47] Kardan MR, Setayeshi S, Koohi-Fayegh R, Ghiassi-Nejad M (2003). Neutron spectra unfolding in bonner spheres spectrometry using neural networks. Radiat. Prot. Dosim..

[48] Griffith, R. V., Palfalvi, J. & Madhvanath, U. Compendium of neutron spectra and detector responses for radiation protection purposes. IAEA Technical Report Series No.318 (Vienna: IAEA) (1990).

[49] Kim YS, Khazaei Z, Ko J, Afarideh H, Ghergherehchi M (2016). Estimation of photoneutron yield in linear accelerator with different collimation systems by Geant4 and MCNPX simulation codes. Phys. Med. Biol..

[50] Hakimi A, Sohrabi M, Mahdavi SR (2017). Photoneutron depth dose equivalent distributions in high-energy X-ray medical accelerators. Phys. Med..

[51] Sohrabi M, Hakimi A (2019). Novel air-to-tissue conversion factors for fast, epithermal and thermal photoneutrons in a Siemens ONCOR dual energy 18 MV X-ray medical linear accelerator. Radiat. Meas..

[52] Kry SF, Howell RM, Salehpour M, Followill DS (2009). Neutron spectra and dose equivalents calculated in tissue for high-energy radiation therapy. Med. Phys..

[53] Sohrabi M, Mostofizadeh A (1999). Measurement of photoneutron doses in and out of high-energy X-ray beam of a SATURNE-20 medical linear accelerator by ECE polycarbonate detectors. Radiat. Meas..

